# Statistics of Hospital and Out-Patient Service

**Published:** 1887-01

**Authors:** 


### STATISTICS OF HOSPITAL AND OUT-PATIENT SERVICE.

### VETERINARY DEPARTMENT. UNIVERSITY OF PENNSYLVANIA, Sept. 1st, 1885, to Sept. 1st, 1886.

| | Hospital. | Out Patient. |
|---|---|---|
| Abscess | 2 | 1 |
| Amputation. Legs dog | 2 | |
| " Tail dog | | 1 |
| " Rudimentary penis. Horse | | 1 |
| Anæmia | | 4 |
| Anasarea | 2 | |
| Arthritis | 3 | |
| Atrophy. Muscular | | 2 |
| Broken knee | 1 | |
| Bronchitis | 1 | 6 |
| " Chronic | | 1 |
| Bruised feet | 2 | |
| " heel | | 2 |
| Burnt sole | 1 | 4 |
| Bursa | 1 | 2 |
| Caries. Superior Maxillary | | 2 |
| Castration | 3 | |
| Cataract | | 2 |
| Catarrh. Auricular | 3 | 6 |
| Champignon | 1 | 1 |
| Chorea | 6 | 1 |
| Cicatrix. Painful | 1 | 3 |
| Colic. Intestinal | 5 | 2 |
| " Obstruction of bowels | | 2 |
| Collection of sinuses | 1 | 11 |
| Congestion of liver | 1 | |
| " spine (Azoturia) | 5 | |
| Conjunctivitis | | 5 |
| " Ulcerated | 1 | |
| Constipation | | 2 |
| Contracted heels | 11 | 20 |
| Contraction of the flexor tendons | 2 | 1 |
| Contusions | 3 | 6 |
| Convulsions | 5 | 1 |
| Corns | 6 | 15 |
| " Suppurating | 2 | 1 |
| Crapandine | 2 | 1 |
| Curb | 1 | 6 |
| Cyst. Serous | 2 | 5 |
| " Testicles | | 1 |
| Degeneration. Fatty liver | 1 | |
| Deafness | 1 | |
| Diarrhœa | 2 | 2 |
| Dislocation of eye | 1 | |
| Distemper. Dog | 18 | 3 |
| Docking | 2 | |
| Dropsy. Abdominial | 1 | |
| Ears. Cut | 1 | |
| Eczema | 6 | 19 |
| Elephantaisis | 2 | 1 |
| Emphysema | | 1 |
| Erythema | | 2 |
| Eventration | 1 | |
| Examination for soundness | | 61 |
| Fistula. Ani | | 1 |
| " Fetlock | | 1 |
| " Withers | 6 | 12 |
| Foreign bodies. Bone in throat | | 1 |
| " " Foot | | 1 |
| " " Ear | | 1 |
| " " Needle in tongue | | 1 |
| Fractures. Ribs | 1 | |
| " Humerus | | 3 |
| " Pelvis | 1 | 1 |
| " Scapula | | 2 |
| " Tibia | | 3 |
| Founder | 1 | 4 |
| " Chronic | 5 | 2 |
| Gastritis | 3 | 4 |
| " Chronic | 2 | |
| Gastro enteritis | 5 | 2 |
| Grease | | 3 |
| Hæmorrhage | | 1 |
| Helminth Ascaridses | 2 | 7 |
| " Tænia | | 2 |
| Hernia. Insigual | | 5 |
| Hoof broken | 1 | |
| Immobility | | 1 |
| Impaction of rectum | 3 | 1 |
| Indigestion. Chronic | 3 | 11 |
| " Intestinal | 2 | 4 |
| Inflammation. Sheath of tendon | 1 | 2 |
| Influenza | 3 | 1 |
| Ingrowing nails | | 1 |
| Interfering | | 1 |
| Keratitis | | 2 |
| Lameness. Shoulder | | 1 |
| Lampas | | 2 |
| Lactation | 1 | |
| " Nervous | | 3 |
| Laryngitis | 1 | 6 |
| " Chronic | 2 | |
| Leucocythæmia | 2 | |
| Locomotor ataxia | 1 | |
| Luxation of hip | | 1 |
| Navicular disease | 3 | 9 |
| Necrosis | 1 | 2 |

| | Hospital. | Out Patient. |
|---|---|---|
| Mange | 18 | 9 |
| Meningitis | 3 | |
| Onychia | | 1 |
| Opthalmia Periodic | | 7 |
| " Traumatic | 6 | 4 |
| Osselettes | 3 | 1 |
| Paralysis | 1 | |
| " Traumatic | | 1 |
| " Lower lip. Melanotic | | 1 |
| " Lumbar. Dog | | 1 |
| " Penis | | 1 |
| Parotiditis | | 1 |
| Pharyngitis | | 1 |
| Phimosis | 1 | |
| Peruositiis | 5 | 2 |
| " Rheumatic | 1 | |
| Pneumonia | 9 | 3 |
| Peritonitis | | 2 |
| Pemphigus | | 1 |
| Pleurisy | 1 | |
| " Traumatic | 1 | |
| " Chronic | 2 | |
| Poisoning. Mercurial | 1 | |
| " Lead | 1 | |
| Poll evil | 4 | 7 |
| Pricked foot | 2 | 7 |
| Prostrate. Enlarged | 1 | 2 |
| Quarter crack | 4 | 8 |
| Quittor | 6 | 3 |
| Ringbone | 11 | 12 |
| Ringworm | 1 | 4 |
| Roaring | 2 | 6 |
| Rupture. Muscular | 1 | 3 |
| Shoe boil | 4 | 6 |
| Shoeing | 2 | 141 |
| Sidesbone | 4 | 7 |
| Spavin | 13 | 17 |
| Spayed. Bitches | 10 | 1 |
| Splints | 3 | 3 |
| Strain of fetlock | 2 | 1 |
| " Ligaments of cervical vertrebra | 1 | |
| " Suspensory ligaments | 1 | 2 |
| Suppurating sole | 1 | |
| Synovitis. Carpal sheath | 8 | 14 |
| " Great sesamoid sheath | 6 | 7 |
| " Hock | 2 | 1 |
| " Patella | 1 | 2 |
| " Femoro coccygeal | | 1 |
| Surfeit | 1 | 6 |
| Teeth. Filed | | 37 |
| " Irregular | | 2 |
| " Removed | | 4 |
| Thrush | 4 | 7 |
| Thumps. Spasm Diaphragm | | 1 |
| Toe Crack | 2 | 3 |
| Tuberculosis | 3 | |
| Trismus | | 1 |
| Tetanus | 2 | |
| Tumors. Carcinoma | | 1 |
| " Epithelioma | 3 | 11 |
| " Fibroma | 4 | 3 |
| " Sarcoma | 1 | 2 |
| " Lypoma | 2 | 1 |
| " Melanotic | 2 | |
| " Myxoma | | 2 |
| " Sebaceous | | 1 |
| " Vasculo-Fibroma | | 2 |
| Ulcers | 4 | 6 |
| Urethritis | | 2 |
| Wounds. Bites | 1 | |
| " Incised | 2 | 2 |
| " Lacerated | 6 | 5 |
| " Surgical | 2 | 1 |
| Diagnosis (unrecorded).* | | |
| Total | 353 | 867 |

*Owing to confusion of a new establishment, a certain number of papers containing records were lost.

*The following cases are selected from the hospital register as offering special features of interest:*

*Nervous Lactation.*—Skye terrier bitch, 6 years old, has never been with male, during the last three years has been in heat several times, and at regular intervals of about four months has had the mammary glands fill with milk. At this period the animal has made her nest, selected an india-rubber ball (the same ball each time from among a number of others), and nursed it for several days, when the lactation ceased. Each lactation was accompanied by an acute gastritis requiring treatment, the last attack, gastro duodenitis, ended fatally. The autopsy showed a liver in advanced stage of fatty degeneration and sclerosis.

*Strain Cervical Ligaments — Simulating Luxation — Syncope from Pain.*—Bay gelding 6 years, 16 hands, work horse, lymphatic temperament. Found cast in stall on near side, with halter strap under off hind foot, head and neck bent in corner of stall. On being loosened got up, little injured except that head and neck were carried to off side, convexity of neck on near side showed bony prominence of transverse processes of first five cervical vertebræ. Attempted reduction by pressure on shoulder and anterior extremity of neck on concave side, counter pressure center of neck on convex side with only partial success. The animal evinced great pain and suddenly fell, entirely unconscious, pulse feeble, and surface cold Recovered consciousness in five minutes, after application of cold water and ammonia.

Application of blister to convex side of neck; no result.

*Lobular Pneumonia.*—Embolism Filaria Immitis (Leidy). Irish setter, 6 years, had distemper in second year, no history of exposure. Broncho-pneumonia both lungs, emaciated, fever moderate, appetite fair, improved slightly under treatment for three weeks, fever and local symptoms unchanged, pleurisy right side, death in five days.

*Autopsy* : Excessive emaciation, lobular pneumonia both lungs, pleurisy right pleura, heart dilated, right ventricle and auricle filled with filaria immitis, at each spot of pneumonia and embolism of two or three to a dozen parasites, a few tubercles formed by encysted parasites, other points degenerated into abscesses.

*Double Penis.*—Gray gelding, 8 years, 16 hands, coach horse, has been unable to protrude penis for two years, purulent discharge from sheath since three months. Examination reveals cavity dorsal surface of penis with evident fibrous band; cast, on drawing out penis found, on dorsal surface at point of reflection of tegument from penis to internal lining sheath, a small rudimentary but perfectly formed penis about the size of a man's. Urethral opening of rudimentary penis leads to duct through entire length ending in cul-de-sac above corpus cavernosum of normal penis. Excessive secretion of sebaceous matter has acted as irritant and and caused abscess. Amputation. Recovery.

*Pelvic Lipoma.*—Recovery. Bay gelding, 7 years, road horse, constipated for four weeks, only passage a fluid mixture in small quantity, three days ago sudden discharge of purulent matter and freer evacuation of excrement. Rectal examination, find above rectum a tumor, size of child's head, opening from upper wall of rectum easily dilated to size of hand and leading to abscess cavity. Enucleated by hand and scissors removing in sections through natural opening. Recovery in three weeks.

*Pelvic Melanosis.*—Death. Gray gelding, 15 years, 15.3 hands, roadster. Melanotic tumors on under surface of tail for several years, tumors right side of anus noticed two months ago, since that time has been constipated, for last ten days no passage beyond slight serous diarrhœa, general depression. Rectal examination shows large irregular tumor size of canteloupe above and to right side of rectum. Incision to right of anus through ischio-anal muscle, enucleation of tumor by hand and scissors, anterior pedicle size of goose egg, hæmorrhage of internal pudic or large branch of it, elbow length from external wound, tied en masse after loss of about four quarts of blood. Little fever and favorable condition for sixty hours, sudden rise of temperature, sensitiveness of abdomen—peritonitis—death.

*Remarks.* Incision would have been better through roof of rectum, and anterior pedicle should have been left, instead of the traction required to remove it which produced the hæmorrhage and brought cavity into immediate contact with peritoneum.

*Sarcomatous Diathesis.* Awakened by castration, gray colt, 2 years, 15.3 hands, model of draft horse. Two small tumors on right inferior thoracic region. Castrated. Healing slow, discharged in five days, returned twelve days from operation with œdema sheath of penis, tumors, size of goose egg on left cord, size of a setter's head on right cord and new formation right side of sheath. Removed tumors of cord from sheath and tumors side of thorax and found all sarcomatous. Wounds healed rapidly. No return during next six months.

*Fracture Femor.* Recovery. Bay gelding, 9 years, 16 hands, common lymphatic work horse, spavin off hock, cast, fired, on getting up showed great lameness and inability to use off hind leg, fracture of femor upper third. Owner refused to abandon, placed animal in slings, but slight febrile reaction, appetite good, removed from slings in seventy-two days, lame, but can use leg, gradual diminution of lameness, in four months visible shortening of leg, but little lameness. Destroyed. Autopsy, comminuted fracture of head of femor and great trochanter, firm moderate callous.

*Fracture Lumbar Vertebræ,* no symptoms for three days, bay gelding, 7 years, 16.1 hands, work horse.

Entered for quittor off hind, cast, operation, got up, noticed slight shaking of tail, but walked to stall same afternoon, the next and third day walked into foot-bath, apparently much better, less lame. On fourth day found on side, refused to get up. Fifth day got up when urged by whip, stands readily, can lift any one foot singly, cannot walk, falls in few minutes. Death. Autopsy shows fracture through body of first lumbar vertebra, hæmorrhage into small psoas. Animal showed no paralysis or anæsthesia of posterior extremities.

*Open Hock Joint Recovery.*—Bay gelding 9 years old, coach horse, a large lacerated wound of the anterior face of the hock joint from a barbed wire fence. Anterior face of the hock a lacerated wound about three inches long in a transverse direction, interesting the anterior attachment of the muscular portion of the flexor metatarsi and the synovial bursa. An abundant discharge of synovia was present, estimated to be at least 5 or 6 oz. in the 24 hours, no purulent infection of the synovial membrane was discovered. The wound after being thoroughly cleansed and disinfected, was dressed with an antiseptic compress dressing, using a narrow flannel bandage thoroughly wet, and applying it as a single spica. This dressing remained about ten days causing a small slough of the skin over the gastrocnemii tendon, but the wound in the hock was closed. It was afterwards treated as an open ulcer, recovery was very prompt, the animal returned to work in 28 days.

*Omental Hernia* (*Traumatic*) *Recovery.*—Bay mare 10 years old, worked in a dump cart, while driving along the street some one called the driver's attention to the fact (to use his own words) "that his horses guts were coming out," he supported the mass by a piece of sacking attached to the shafts of the cart and in this way drove to the hospital. An examination showed a punctured wound of the abdominal walls inferiorly, a few inches posterior to the costal cartilages; from which had escaped a mass of omentum as thick as the three fingers and over two feet long. Two large pins were passed through the lips of the wound and the omental mass, a figure of 8 ligature applied and the omentum cut off close to the belly, a mustard plaster was now applied to the whole of this region causing, as was intended, a large œdematous swelling. The animal never showed the slightest unfavorable symptom and returned to work in five days.

